# A New Nuclear Function of the *Entamoeba histolytica* Glycolytic Enzyme Enolase: The Metabolic Regulation of Cytosine-5 Methyltransferase 2 (Dnmt2) Activity

**DOI:** 10.1371/journal.ppat.1000775

**Published:** 2010-02-19

**Authors:** Ayala Tovy, Rama Siman Tov, Ricarda Gaentzsch, Mark Helm, Serge Ankri

**Affiliations:** 1 Department of Molecular Microbiology, The Bruce Rappaport Faculty of Medicine, Technion, Haifa, Israel; 2 Department of Chemistry, The Pharmacy and Molecular Biotechnology Institute, Ruprecht-Karls University of Heidelberg, Heidelberg, Germany; 3 The Pharmacy and Biochemistry Institute, Johannes Gutenberg University, Mainz, Germany; University of Virginia Health System, United States of America

## Abstract

Cytosine-5 methyltransferases of the Dnmt2 family function as DNA and tRNA methyltransferases. Insight into the role and biological significance of Dnmt2 is greatly hampered by a lack of knowledge about its protein interactions. In this report, we address the subject of protein interaction by identifying enolase through a yeast two-hybrid screen as a Dnmt2-binding protein. Enolase, which is known to catalyze the conversion of 2-phosphoglycerate (2-PG) to phosphoenolpyruvate (PEP), was shown to have both a cytoplasmatic and a nuclear localization in the parasite *Entamoeba histolytica*. We discovered that enolase acts as a Dnmt2 inhibitor. This unexpected inhibitory activity was antagonized by 2-PG, which suggests that glucose metabolism controls the non-glycolytic function of enolase. Interestingly, glucose starvation drives enolase to accumulate within the nucleus, which in turn leads to the formation of additional enolase-*E.histolytica* DNMT2 homolog (Ehmeth) complex, and to a significant reduction of the tRNA^Asp^ methylation in the parasite. The crucial role of enolase as a Dnmt2 inhibitor was also demonstrated in *E.histolytica* expressing a nuclear localization signal (NLS)-fused-enolase. These results establish enolase as the first Dnmt2 interacting protein, and highlight an unexpected role of a glycolytic enzyme in the modulation of Dnmt2 activity.

## Author Summary

Epigenetics refers to heritable changes in gene function that occur without alterations in the DNA sequence. The best characterized epigenetic modification is DNA methylation. In mammals, DNA methylation is associated with gene silencing and transposon control. We have previously established the presence of methyl cytosine in the genome of *Entamoeba histolytica*, an important unicellular human pathogen. Ehmeth, an enzyme that belongs to the DNA methyltransferase 2 (Dnmt2) family, catalyzes DNA methylation in the parasite. Recent evidence in support of the notion that human Dnmt2 is a tRNA^Asp^ methyltransferase fuels the debate about the real function of the Dnmt2 family. Our results show that Ehmeth also catalyzes tRNA^Asp^ methylation and indicates a dual function for this protein. In this study, we have also identified that enolase, a glycolytic enzyme, interacts with Ehmeth, and modulates its activity under conditions of glucose starvation. These data add to the emerging evidence that glycolytic enzymes have multifunctional roles, and emphasize the importance of energetic metabolism in the control of the epigenetic enzymatic machinery.

## Introduction

The synthesis of 5-methylcytosine in both DNA and RNA is catalyzed by methyl 5-cytosine methyltransferases (m5C-MTase) with *S*-adenosylmethionine as a cofactor. The mammalian DNA methylation machinery consists of three active DNA m5C-MTases: Dnmt1, Dnmt3a and Dnmt3b. Dnmt1 has a high preference for hemi-methylated DNA as a substrate [1], whereas Dnmt3a and Dnmt3b are *de novo* DNA MTases that act on non-methylated DNA (for review, see Jeltsch [2]). A fourth DNA m5C-MTases, Dnmt2, belongs to a large family of proteins that are conserved in all species from *Schizosaccharomyces pombe* to humans. Dnmt2 stands apart from the three active DNA MTases because its length is relatively short when compared to that of Dnmt3a, Dnmt3b, or Dnmt1. Furthermore, this enzyme resembles prokaryotic DNA MTases because it does not have a large N-terminal regulatory domain [3].

Native tRNA^Asp^ extracted from Dnmt2-deficient mice, *Arabidopsis thaliana* or *Drosophila melanogaster* were methylated *in vitro* by the human Dnmt2 (hDnmt2) protein. Accordingly, it was proposed that hDnmt2 is a tRNA^Asp^ MTase rather than a DNA MTase [4], an idea that was further supported by the fact that it can also methylate transcribed tRNAs in vitro [5],[6]. On the other hand, the role of Dnmt2 seems to be not essential in higher eukaryotes because loss of function mutations of the Dnmt2 gene do not change genomic methylation patterns in the mouse [7]. In addition, depletion of *D. melanogaster* Dnmt2 (dDnmt2) by RNA interference has no detectable consequences on embryonic development [8]. However, a recent report has shown that loss of Dnmt2 in somatic cells eliminates H4K20 trimethylation at retrotransposons, and impairs maintenance of retrotransposon silencing [9]. Dnmt2 has been established as a genuine DNA methyltransferase in lower eukaryotes. Dnmt2 catalyzes DNA methylation in *Dictyostelium discoideum* [10],[11] and *Entamoeba histolytica* [12]. However, the weak DNA methyltransferase activity and the low expression level of Dnmt2 enzymes may explain the low methylation level that is found in these organisms [13]. Dnmt2 catalyzes cytosine methylation with a low preference for Cp(A/T) [8],[12],[14] or CC(A/T)GG [15], rather than the CpG motif. These results suggest that a dual specificity for DNA and RNA substrates emerged during the evolution of the Dnmt2 family [13]. Despite this dual specificity for DNA and RNA, the function of Dnmt2 as an RNA methyltransferase in lower eukaryotes has not yet been established.

The finding of interacting partners to members of the DNA/tRNA methyltransferase Dnmt2 is crucial for improving our existing understanding of its function. Until now, no interacting candidate has been reported for this family of proteins. In contrast, numerous proteins have been shown to interact with Dnmt1 and Dnmt3 thereby linking methylation to histone modifications and transcription regulation. For example both Dnmts were found to be associated with histone deacetylase [16],[17]. Dnmt1 was also found to interact with several chromatin- associated proteins, such as retinoblastoma protein, DNA methyltransferase 1 associated protein 1 and methyl CpG binding protein 2 [1], and Dnmt3 binds various transcription regulators, such as the transcriptional regulator RP58, the fusion protein of promyelocytic leukemia (PML) and the retinoic acid receptor-α (RARα) (PML-RAR) and heterochromatin protein 1 [18].

*E.histolytica* is an interesting model in which to study DNA methylation because Ehmeth, an enzyme that belongs to the Dnmt2 family, is the unique DNA methyltransferase that is present in this parasite [12]. The presence of methylated cytosine in *E. histolytica* ribosomal DNA [12] and the scaffold/matrix attachment region [19], together with the evidence that mutations can result from accelerated deamination of methylated cytosines in the reverse transcriptase of LINE retrotransposon (RT LINE) [20] support a role for Dnmt2 in the control of repetitive elements. This role has been confirmed in lower eukaryote *Dictyostelium discoideum* [10],[11] and in *Drosophila* [9]. Here, we establish that Ehmeth can catalyze the methylation of tRNA^Asp^. Moreover, we report, for the first time, that enolase, in addition to its involvement in the glycolytic pathway [21],[22], is an inhibitor of Dnmt2.

## Results

### Identification and validation of enolase as an interacting partner of Ehmeth

We carried out a yeast two-hybrid screen using a bait vector that expressed pAS1-Ehmeth that was fused to the GAL4 binding domain (GAL4BD) and an *E.histolytica* cDNA library that was fused to the GAL4 activation domain (GAL4AD) as prey. For this purpose, 10^6^ clones were analyzed, and only two were selected based on their ability to grow on the selective medium (histidine, leucine, tryptophan and adenine) and results from the β-galactosidase complementation assays (data not shown). For each of the two positive clones, the recombinant plasmid that harbored the cDNA sequence that was fused to GAL4AD was isolated by transformation of *E*. *coli* cells, and then sequenced. These plasmids encode alcohol dehydrogenase (Accession number xp_653507.1) and enolase (Accession number xp_649161.1), respectively. Alcohol dehydrogenase was excluded from our analysis due to the presence of a frame shift mutation in its sequence.

**Figure 1:**
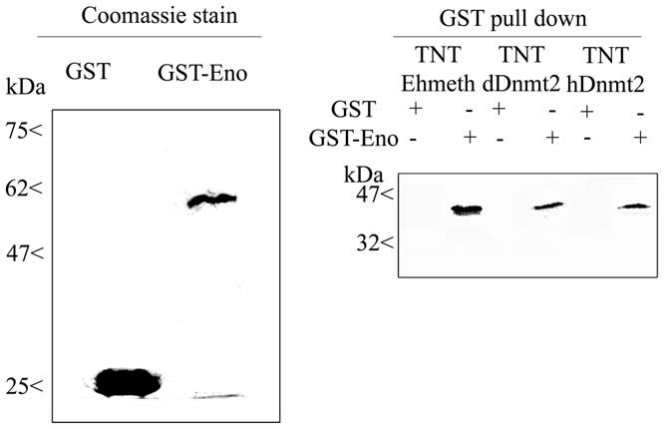
*In vitro* interaction between Ehmeth, dDnmt2, hDnmt2 and enolase. ^35^S labeled proteins Ehmeth (TNT-Ehmeth), dDnmt2 (TNT-dDnmt2) and hDnmt2 (TNT-hDnmt2) were incubated respectively with glutathione beads coated with GST or GST- Enolase and the interacting proteins were analyzed by SDS-Page as described in the *Materials and Methods*. Left panel: Coomassie staining of GST and GST-Enolase fusion protein used in the pull-down procedure. Right panel: Pull down products TNT-Ehmeth, TNT-dDnmt2 and TNT-hDnmt2 were detected by exposure of the membrane to an x ray film.

In order to validate the interaction between enolase and Ehmeth, we carried out GST pull-down experiments. Ehmeth was first transcribed *in vitro*, and then translated in the presence of radioactive 35-S-methionine (TNT system) before incubating it with gluthatione beads that were coated with either GST-Ehenolase or GST. The result of this pull-down experiment shows that Ehmeth binds specifically to GST-Ehenolase, and not to GST (Fig. 1).

The existence of sequence homology between members of the Dnmt2 protein family and members of the enolase family suggests that the interaction between Ehmeth and enolase is conserved outside the *Entamoeba* genus. In order to test this hypothesis, *Drosophila* and human Dnmt2 proteins were transcribed in vitro, translated, and then incubated with GST-Ehenolase. Interestingly, both Dnmt2 proteins were able to bind to enolase (Fig. 1).

### Localization of enolase in *E.histolytica* trophozoites

**Figure 2:**
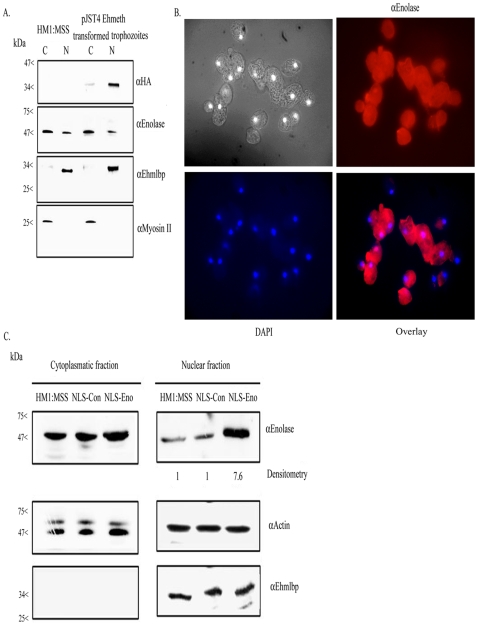
Enolase is present in the cytoplasmatic and nuclear fraction of *E.histolytica*. A. Cytoplasmatic (C) and nuclear (N) protein fractions of *E.histolytica* HM-1∶MSS and pJST4-Ehmeth trophozoites were separated on 12% SDS-PAGE and analyzed by western blot with an anti HA antibody, an anti enolase antibody, an anti EhMLBP antibody or an anti Myosin II antibody. B. Cellular localization of Ehenolase in *E.histolytica* trophozoites. Ehenolase was detected by immunofluorescence microscopy using anti-enolase antibody. Ehenolase distribution is shown in red using a primary anti-enolase antibody and a secondary antibody conjugated with Cy3. Nuclei (blue) were stained by DAPI. Computer-assisted image overlay analysis of the signal given by enolase antibody and by DAPI, shows that Ehenolase is ubiquitously present in trophozoites including in the nucleus. C. Cytoplasmatic and nuclear protein fractions of *E.histolytica* HM-1∶MSS, trophozoites expressing a NLS-fused-scramble peptide (NLS-Con) (30) and trophozoites expressing a NLS-fused enolase (NLS-Eno) were separated on 12% SDS-PAGE and analyzed by western blot with an anti enolase antibody, an anti actin antibody or an anti EhMLBP antibody.

We previously reported that enolase is secreted by activated trophozoites [23]. In order to get further insights into the cellular localization of this protein, cytoplasmatic and nuclear trophozoite proteins that were prepared from HM-1∶MSS trophozoites were analyzed by western blotting with an antibody against enolase (Fig. 2A, 2C). The specificity of the enolase antibody that was raised against human enolase was confirmed against GST-Ehenolase using GST alone as the negative control (data not shown). The efficiency of the protein fractionation was examined by western blot analysis using antibodies against EhMLBP, a nuclear protein [24] and myosin II, a cytoplasmatic protein [25], as controls. As expected, EhMLBP was detected in the nuclear fraction and Myosin II in the cytoplasmatic fraction of the parasite (Fig. 2A). Enolase was detected as a 47 kDa protein present in the cytoplasmatic fraction of the parasite (Fig. 2A, 2C). Moreover, non-negligible amount of enolase were detected in the nuclear fraction of the parasite. To further validate these results, we examined the localization of enolase in the parasite using immunofluorescent microscopy (Fig. 2B). The result of this analysis showed that enolase is ubiquitously present in the parasite including its nucleus.

### Ehmeth interacts with enolase in *E.histolytica in vivo*

In order to test the binding of Ehmeth to enolase in the parasite, we conducted co-immunoprecipitation experiments using endogenous enolase with a calmodulin, histidine, hemagglutin (CHH)-tagged-Ehmeth in pJST4-Ehmeth transfected trophozoites nuclear lysate. We chose a tagged Ehmeth rather than the endogenous Ehmeth in these co-immunoprecipitation experiments because the antibody that we previously raised against Ehmeth [12] was unable to immunoprecipitate the protein (data not shown). A hemagglutin (HA) antibody was used to detect HA in the CHH tag. The expression of CHH-tagged Ehmeth in the nuclear fraction of pJST4-Ehmeth transfected trophozoites was confirmed by western blot analysis using an HA antibody (Fig. 2A).

**Figure 3:**
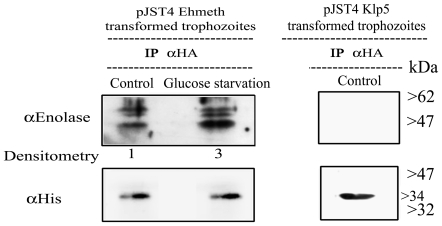
*In vivo* interaction of Ehmeth with enolase. Immunoprecipitation with an anti-HA antibody from a nuclear lysate of *E. histolytica* trophozoites that express Ehmeth as a CHH-tagged protein (pJST4-Ehmeth) grown in regular media (control) and from trophozoites grown in a glucose starvation media (glucose starvation). Detection of immunoprecipitated proteins was done by western blot with an anti-enolase antibody. To validate that the same amounts of Ehmeth were used in the assay, immunoprecipated proteins were analyzed with an anti His antibody which detects the CHH tagged Ehmeth. As a negative control, immunoprecipitation with an anti-HA antibody from a nuclear lysate of *E. histolytica* trophozoites that express CHH-klp5 was used (right panel). The physical interaction between enolase and Ehmeth is demonstrated only after immunoprecipitation from Ehmeth tagged trophozoites and this complex is enhanced following glucose starvation (3 fold according to Tina densitometry analysis).

We observed that enolase co-immunoprecipitated with CHH-tagged-Ehmeth (Fig. 3 left panel, Control). Ehmeth also co-immunoprecipitated with enolase (data not shown). In order to exclude the possibility that enolase interacts with the CHH tag and not with Ehmeth, enolase was immunoprecipitated from a nuclear lysate of trophozoites that expressed a CHH-KLP5 tagged protein [26] using the HA antibody. We observed that enolase does not co-immunoprecipitate with the CHH-KLP5 tagged protein, and this result indicates that no interaction occurred between enolase and the CHH tag (Fig. 3, right panel).

### Mapping of Ehmeth binding site to enolase

**Figure 4:**
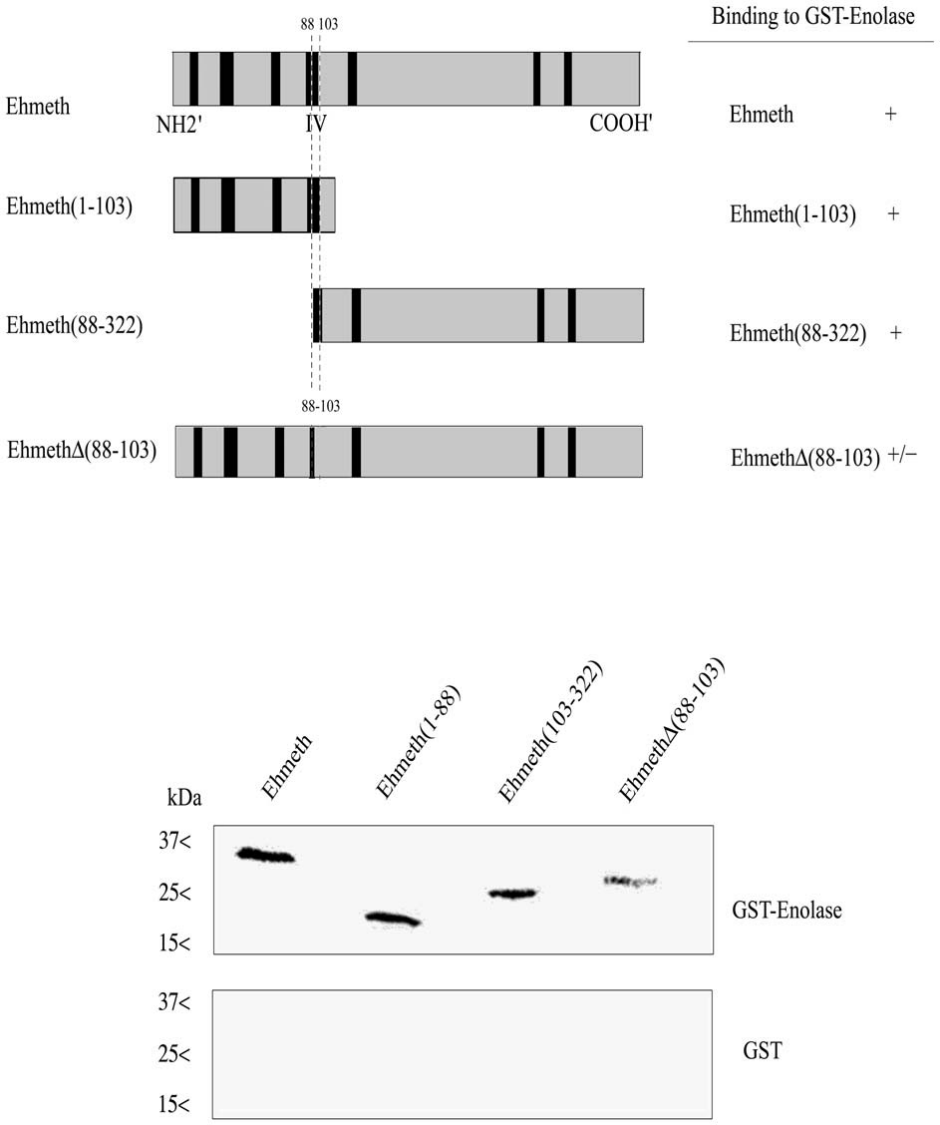
Mapping of the enolase binding region of Ehmeth. Upper panel: Scheme of the different Ehmeth mutants. Lower panel: Pull down experiment of different Ehmeth fragments with recombinant enolase. Whereas Ehmeth full length, Ehmeth (from amino acid 1 to103) and Ehmeth (from amino acid 88 to 322) where efficiently pull-down by enolase, Ehmeth that has its domain IV truncated interacts poorly with enolase.

In order to delineate the enolase-interacting domains on Ehmeth, a series of deletion mutant proteins (Fig. 4, upper panel) were pulled down by either GST-Ehenolase or GST. We observed that N-terminal (from amino acid 1 to 103) and C-terminal (from amino acid 88 to 322) of Ehmeth were able to bind enolase in the same manner as full length Ehmeth (Fig. 4 lower panel). These results suggest that the specific region between amino acid 88 and 103, which is shared by the C-terminal and N-terminal Ehmeth mutant proteins is involved in the binding of Ehmeth to enolase. This region includes the catalytic site (domain IV) of Dnmt2 proteins [27]. In order to test this hypothesis, a mutant Ehmeth protein that lacks the amino acids 88 to 103 (EhmethΔ88–103) was generated, and its binding to GST-Ehenolase was examined. We found that the binding of EhmethΔ88–103 to enolase is impaired (Fig. 4 lower panel). It is important to emphasize that the input amount of the different Ehmeth deletion mutants proteins used in the GST-pull down assay were equivalent (data not shown). This result indicates that the domain IV contributes to the binding of Ehmeth to enolase. The catalytic domain of Dnmt2 proteins subsists as an exposed loop which is not part of the main structure [3]. According to this model, no significant conformational change in the structure of Ehmeth is expected , following the deletion of the amino acids 88 to 103.

### Enolase inhibits the binding of Ehmeth and hDnmt2 to EhMRS2 DNA

**Figure 5:**
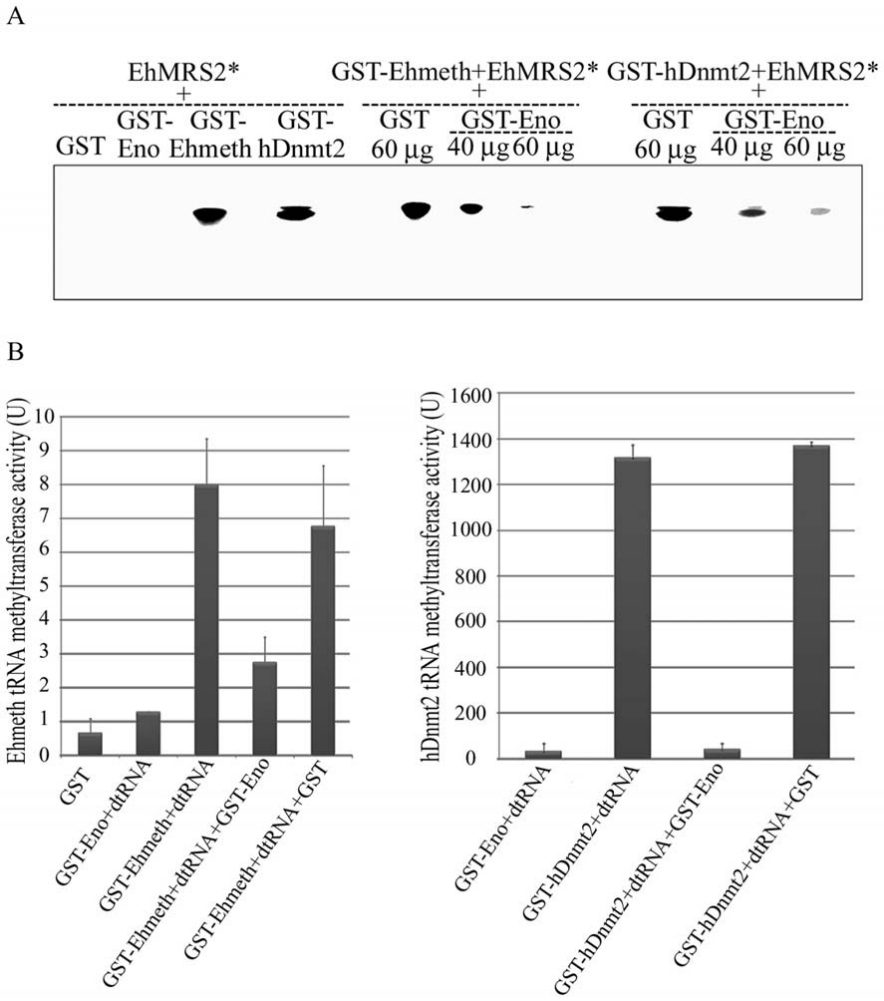
Enolase inhibits Ehmeth and hDnmt2 functions. A. The binding of γ−ATP labeled EhMRS2 DNA (EhMRS2*−0.33 µg) to Ehmeth or hDnmt2 was detected as a DNA-protein complex. No complex was observed between GST and GST-Enolase incubated with EhMRS2 DNA. Enolase inhibits the binding of Ehmeth and hDnmt2 to EhMRS2 DNA in a dose dependent manner. B. Effect of enolase on the Ehmeth (left panel) and hDnmt2 (right panel) tRNA methyltransferase activity. The results represent the mean and standard deviation of three independent experiments (P_value_<0.05). U = one unit corresponds to 1 pmol of H^3^-Adomet incorporated/hour/nmol of enzyme.

We previously demonstrated that Ehmeth binds to EhMRS2, a DNA element, which contains the eukaryotic consensus scaffold/matrix attachment regions (S/MAR) bipartite recognition sequences [19]. We hypothesized that enolase regulate Ehmeth activity because it binds to its catalytic site. In order to test this hypothesis, GST-Ehmeth was incubated with P^32^ labeled EhMRS2 DNA in presence of various amount of GST-Ehenolase, and the denaturant-resistant DNA-Ehmeth complex [3] was analyzed by SDS-PAGE under denaturing conditions. In agreement with a previous report [19], GST-Ehmeth forms a complex with EhMRS2 DNA which is characterized by a retarded band in the SDS gel (Fig. 5A). No complex was observed when the labeled EhMRS2 DNA probe was incubated with either GST or GST-Ehenolase (Fig. 5A). The presence of Ehmeth in the retarded band was confirmed by mass spectrometry analysis (Fig. S1). Remarkably, the formation of Ehmeth-EhMRS2 complex was inhibited in the presence of Ehenolase (Fig. 5A). In order to confirm this result for hDnmt2, we tested its ability to bind EhMRS2 DNA. We found that hDnmt2 binds to EhMRS2 DNA (Fig. 5A). The formation of hDnmt2-EhMRS2 DNA complex was also strongly inhibited by Ehenolase. These results suggest that an identical inhibitory mechanism is used by enolase to inhibit the binding of Ehmeth and hDnmt2 to EhMRS2 DNA.

### Enolase inhibits the tRNA^Asp^ methyltransferase activity of Ehmeth and hDnmt2

It has been reported that hDnmt2 catalyzes the methylation of tRNA^Asp^ [4],[5],[6]. Therefore, we decided to examine this catalytic activity in *E.histolytica* because it has not yet been investigated in unicellular organisms. We found that the catalytic activity for Ehmeth was 9 U (Fig. 5B, left panel). This activity is substantially lower (about 100-fold) than that of hDnmt2 (Fig. 5B, right panel). GST has no detectable tRNA^Asp^ MT activity. It has been reported that hDnmt2 methylates tRNA^Asp^ using a DNA methyltransferase-like catalytic mechanism [6]. This last observation predicts that enolase will also inhibit the tRNA^Asp^ MT activity of Ehmeth and hDnmt2. We confirmed this prediction by showing that the activity of Ehmeth and hDnmt2 tRNA^Asp^ MT was strongly inhibited by enolase (approximately 60% and 90% inhibition, respectively) (Fig. 5B).

### Effect of 2-phosphoglycerate (2-PG) on the inhibitory activity of enolase

**Figure 6:**
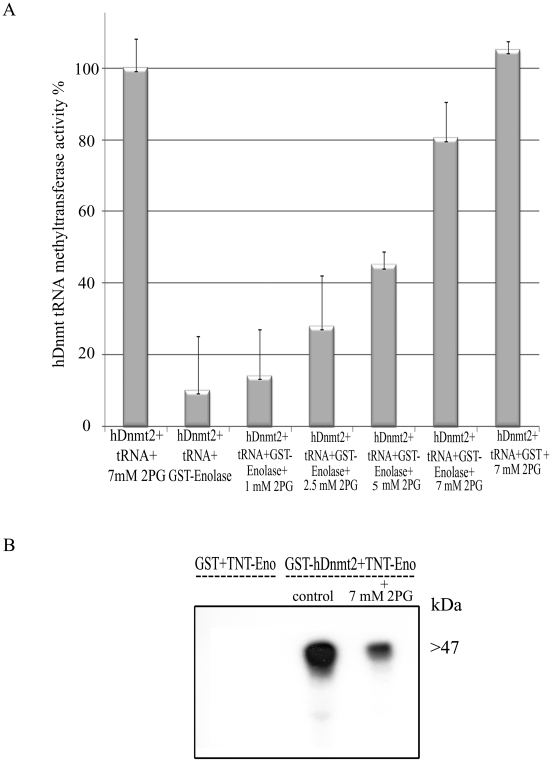
The influence of 2-PG on enolase inhibitory effect over Dnmt2 tRNA MT activity. A. Measure of the hDnmt2 tRNA methyltransferase activity in presence of enolase and increasing concentrations of 2 phosphoglycerate (2-PG). The activity of hDnmt2 measured in the presence of 7 mM 2-PG was regarded as 100%. As already reported enolase strongly inhibits hDnmt2 in absence of 2-PG. The activity of hDnmt2 in presence of enolase is restored by 2-PG in a dose dependent manner. The results represent the mean and standard deviation of three independent experiments (P_value_<0.05). B. *In vitro* interaction between hDnmt2 and enolase in the presence of 7 mM 2-PG. ^35^S labeled enolase (TNT-Eno) was incubated respectively with glutathione beads coated with GST or GST- hDnmt2 in presence or absence of 2-PG (7 mM). The pull down products was detected by exposure of the membrane to an x ray film. According to Tina densitometry analysis around 4 times less Enolase was pull down by hDnmt2 when 2-PG was present in the reaction.

Enolase has been reported to undergo a conformational change following its binding to 2-PG [28],[29]. This observation prompted us to examine the effect of 2-PG on the inhibitory activity of enolase. For this purpose, the ability of enolase to inhibit the methylation of tRNA^Asp^ by hDnmt2 was investigated in the presence of increasing concentrations of 2-PG. For this experiment, hDnmt2 was preferred to Ehmeth because its tRNA MT activity is significantly higher (see Fig. 5B). We observed that the inhibitory activity of enolase was reduced by 2-PG in a dose-dependent manner (Fig. 6A). This result may be explained by reduced enolase binding to hDnmt2 when 2-PG is present. In order to test this hypothesis, the binding of enolase and hDnmt2 was investigated in the presence of 2-PG (7 mM). Following the addition of 2-PG, we observed that the binding of enolase to hDnmt2 was strongly reduced (Fig. 6B). These results indicate that the inhibitory activity of enolase is regulated by its substrate, and suggest a link between the glycolytic pathway and Dnmt2 activity.

### Effect of glucose starvation on the localization of enolase, its binding to Ehmeth and on the DNA/tRNA^asp^ methylation status

Our previous results indicated that 2-PG modulates the inhibitory activity of enolase. In order to assess the physiological relevance of this observation, we used glucose starvation as a means to reduce the level of 2-PG in the parasite. We chose to quantify intracellular pyruvate, the end product of glycolysis, as the method to monitor the effect of 12-hour glucose starvation instead of a direct measurement of 2-PG because its determination is easier than 2-PG. We observed that the level of pyruvate in glucose-starved trophozoites for12 hours was reduced by 50% when compared to non-starved control trophozoites (8×10^−14^ mol/ml vs 8×10^−7^ mol/ml). Longer glucose starvation (24 hours) resulted in significant death of the parasite (more than 50% of the original population, data not shown).

**Figure 7:**
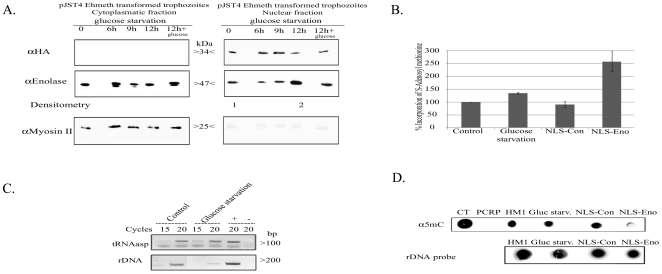
The effect of enolase accumulation in the nucleus over Dnmt2 tRNA and DNA MT activity. A. Western blot analysis of cytoplasmatic and nuclear protein fractions prepared from *E.histolytica* pJST4-Ehmeth trophozoites grown without glucose for (0, 6, 9 and 12 hours or for 12 hours of starvation followed by 12 hours of growth in presence of 1% glucose). Proteins were separated on 12% SDS-PAGE and analyzed by western blot with an anti HA antibody, an anti enolase antibody, or an anti Myosin II antibody. This figure is representative of at least three independent experiments. B. Effect of glucose starvation and continuous forced expression of enolase in the nucleus on the level of tRNA methylation in the parasite. RNA samples from trophozoites grown in regular (control), glucose starvation media (glucose starvation), NLS-Con trophozoites and NLS-Eno trophozoites were used as substrates for in vitro tRNA methylation assay performed with hDnmt2 (see materials and methods). The amount of methyl group incorporate in control RNA was taken as 100%. The significant higher amount of methyl group incorporated in RNA prepared from glucose starved trophozoites (38% increases) and NLS-Eno trophozoites (250% increases) indicates the tRNA present in this sample were less methylated. The results represent the mean and standard deviation of three independent experiments (P_value_<0.05). C. RT PCR analysis of the tRNA^asp^ amount in trophozoites grown in regular (control) and glucose starvation media (glucose starvation). The amount of rDNA was used for the normalization of the data. D. Effect of glucose starvation and continuous forced expression of enolase in the nucleus on the level of m5C methylation in the parasite. Genomic DNA was prepared from trophozoites grown in regular (control), glucose starvation media (glucose starvation), NLS-Con trophozoites and NLS-Eno trophozoites and dot blotted on nitrocellulose membrane in the indicated amounts. Genomic DNA from calf thymus (CT) or PCR product (PCRP) were used as positive and negative controls respectively. DNA methylation was detected with an antibody directed against 5-methylcytosine (α5mc) and the total amount of DNA was estimated by hybridization with a radioactive probe against rDNA.

The localization of enolase during glucose starvation was followed by western blot analysis of cytoplasmatic and nuclear lysates. We consistently observed that at least three times more enolase was present in the nuclear lysate of 12-hour glucose-starved trophozoites than in non-starved control trophozoites (Fig. 7A, right panel). No accumulation of enolase in the nucleus was observed in trophozoites exposed to heat shock or oxidative stress (data not shown). The addition of glucose to the starved parasite restored the original distribution of enolase. This result emphasizes that the mechanism used to accumulate enolase in the nucleus is reversible. Moreover, immunoprecipitation analysis of the enolase-Ehmeth complex following glucose starvation for 12 hours showed that more enolase-Ehmeth complex was formed in the starved trophozoites than in the non-starved control trophozoites (Fig. 3, left panel).

In this study we showed that enolase inhibits Ehmeth. Accordingly, we hypothesized that the formation of Enolase-Ehmeth complex affects the level of DNA and tRNA^Asp^ methylation following glucose starvation of the parasite. In order to test this hypothesis, the level of tRNA and DNA methylation in control and glucose starved trophozoites was determined. Accordingly, we observed, a significant decrease in tRNA methylation (38%) in glucose-starved trophozoites when compared to that determined in the non-starved trophozoites (Fig. 7B). Moreover, RT PCR analysis showed no significant difference in the amounts of tRNA^Asp^ in glucose- starved and non-starved control trophozoites (Fig. 7C). In contrast, when we examined the level of DNA methylation in genomic DNA of control and glucose-starved parasites with an m5C antibody using dot blot analysis we could not detect any differences (Fig. 7D) [12]. This result indicates that DNA methylation is not affected by glucose starvation probably due to the short time (12 hours starvation). Therefore, to further examine the effect of enolase accumulation in the nucleus on DNA methylation we expressed enolase constitutively followed by a Nuclear Localization Signal (NLS) in the parasite.

### Effect of enolase accumulation in the nucleus on DNA and tRNAasp methylation

The transfected trophozoites with NLS Enolase and trophozoites expressing a random 12 amino acids peptide followed by a NLS [30] which were used as control (NLS-Con transfectants) were cultured continuously in the presence of 24 µg mL^−1^ G418 for one month. The localization of enolase in NLS-Eno and NLS-Con transfectants was followed by western blot analysis of cytolasmic and nuclear lysates (Fig. 2C). We observed that 7 times more enolase was present in the nucleus of NLS-Eno transfectants than in NLS-con transfectants or non-transfected trophozoites (HM1∶MSS) (Fig. 2C, right panel). The level of DNA and tRNA^Asp^ methylation in NLS-Con and NLS-Eno was determined (Fig. 7B and D). A significant decrease in both DNA and tRNA^Asp^ methylation was observed in NLS-Eno transfectants when compared to that determined in NLS-Con transfectants. These results indicate that the continuous accumulation of enolase in the nucleus inhibit both Ehmeth DNA and tRNA^Asp^ MT activity.

## Discussion

Of members of the Dnmt family of proteins, the roles of Dnmt1 and Dnmt3 are relatively well understood. In contrast, our knowledge about Dnmt2 is scanty. Furthermore there is no information about the molecules which interact with this protein. Therefore, the identification of such molecules would be a key step towards elucidating our understanding of Dnmt2 functions. Enolase, a glycolytic enzyme that catalyses the conversion of 2-PG to phosphoenolpyruvate, (PEP) is to the best of our knowledge the first Dnmt2-interacting protein to be described. For many years, glycolytic enzymes have been considered to be housekeeping cytoplasmatic proteins. Based on the results of studies on the function(s) of the glyceraldehyde-3-phosphate dehydrogenase, this concept has changed, and it is now well accepted that some of these enzymes that includes enolase, are multifunctional proteins which are involved in gene transcription, DNA replication, DNA repair, and nuclear RNA export (for review see [31]). The inability to select in complex growth media mutants of *Bacillus subtilis* [32], *Escherichia coli* [32] and *E.histolytica* enolase (data not shown) supports this multifunctional role. The catalytic activity of enolase in *E. histolytica* has been characterized [22], and it was found to be co-secreted with serpin and aldehyde alcohol dehydrogenase by activated trophozoites [23]. Indeed, antibodies against enolase have been detected in patients with amebiasis, and this suggests that enolase plays a role in the virulence of the parasite [33]. Such a role has been already reported in bacteria where enolase binds plasminogen [34]. The results of this investigation show that enolase is present in the cytoplasm and nucleus of *E.histolytica*. This ubiquitous localization is not unique to *E. histolytica*. In mammals, there are three isoforms of enolase (for review [35]), and each is characterized by its tissue distribution and expression. In HeLa cells, *A. thaliana*, and *Plasmodium yoelii*, enolase was found also in the nucleus. These observations raise the question about the significance of enolase presence in the nucleus. The results of our investigations on the nuclear role of enolase suggest that it is a Dnmt2 inhibitor.

The results from several recent studies have fuelled the debate on whether Dnmt2 is a DNA methyltransferase, a tRNA methyltransferase, or both. The results of our investigation support the notion that *E.histolytica* Dnmt2 (Ehmeth) is a DNA methyltransferase and a tRNA methyltransferase. Indeed, this is the first report of Dnmt2 being a tRNA methyltransferase in lower eukaryotes. Enolase has been reported to bind the bacteriophage-specific DNA adenine methyltransferase M.EcoT1. Interestingly, enolase binding to M.EcoT1 did not influence M.EcoT1 catalytic activity [36]. The domain IV of Ehmeth includes the catalytic sites, and is widely conserved among DNA-(*cytosine*-C 5)-methyltransferase. The binding of enolase to the domain IV of Ehmeth is probably the main mechanism of its inhibitory action. Dnmt2 methylates tRNA using a DNA methyltransferase-like catalytic mechanism [6]. Therefore, it is not surprising that the binding of enolase to Ehmeth interferes with both EhMRS2 DNA recognition and tRNA^Asp^ MT activity. In *S. cerevisiae*, enolase interacts with cytosolic tRNA^Lys^ in order to enable its translocation into the mitochondria, thereby displaying a function as a tRNA chaperone [37]. Our data showed that enolase does not interact with either DNA or tRNA^Asp^, thereby excluding competition as a mechanism to explain its Dnmt2 inhibitory activity. Only a few proteins have been reported to interact with the C-terminal domain, which contains the catalytic site for Dnmts. The P23 protein is a protein that is associated with steroid receptor complexes binds to the C-terminal of Dnmt1 [38]. However, its effect on Dnmt1 activity is still unclear. In contrast, p53 has been shown to stimulate Dnmt1 activity in vitro by binding to the C-terminal of Dnmt1 [39]. This last example together with our findings reinforce the notion that catalytic activity of Dnmt protein can be modulated by proteins that interact with their C-terminal.

The accumulation of enolase in the nucleus and the formation of an additional Ehmeth-enolase complex following glucose starvation support a central role for glucose metabolism in the regulation of Ehmeth activity. Glucose starvation was preferred to drugs in order to inhibit glycolysis because (i) one of the unwanted action of such drugs is the inhibition of proteasome activity [40], and (ii) the physiological relevancy of glucose starvation during *Entamoeba* differentiation [41]. Metabolites can act as sensors of the cell energy status. Therefore, they are convenient regulators of enzymes under conditions of physiological stress such as glucose starvation. For example, glucose starvation affects the activation or silencing of rRNA expression [42].

Glucose starvation led to significant Trna^Asp^ demethylation, but not to DNA demethylation. In contrast, forced expression of enolase in the nucleus led to both DNA and tRNA^Asp^ demethylation. In mammals, active DNA demethylation is controversial [43]. Recently, a convincing mechanism of active DNA demethylation in which DNA glycosylase act as DNA demethylases through a base-excision-repair pathway has been proposed [44]. There is no evidence that active DNA demethylation occurs in *E.histolytica*. Passive demethylation occurs when DNA methylation is progressively reduced with cell division [45]. The generation time of the parasite is eight hours, and this would make it unlikely that DNA demethylation will occur following 12 hours of glucose starvation. However, this passive mechanism of DNA demethylation has probably occurred in the enolase-NLS strain during the numerous divisions of this strain. In contrast, the turnover of tRNA is much faster, and allows for rapid passive demethylation [46]. The physiological meaning of the Dnmt2-mediated methylation on tRNA^Asp^ is still unknown. tRNA methylation has been involved in the control of tRNA stability [47],[48]. In *S. cerevisiae*, Trm9 mediated tRNA methylation is linked to the translation enhancement of genes related to stress response, DNA damage and other cellular functions [49],[50]. Mitochondrial tRNA methylation mediated by Trm 5 was shown to regulate mitochondrial protein synthesis [51]. These different functions for tRNA methylation represent an interesting starting point for further research on the role of tRNA^Asp^ methylation in *E.histolytica*.

To conclude, the results of this investigation provide *in vivo* and *in vitro* evidence that establishes enolase as the first Dnmt2 interacting protein. Moreover, our results also provide strong evidence that link glucose metabolism and Dnmt2 activity. In addition, we have also shown that Dnmt2 is a tRNA methyltransferase in lower eukaryotes. The question of the significance of enolase-Dnmt2 interaction is higher eukaryotes needs further investigation.

## Materials and Methods

### Microorganisms used in this study

Trophozoites of the *E. histolytica* strain HM-1∶IMSS were grown under axenic conditions in Diamond's TYI-S-33 medium (glucose concentration 750 mg/l) at 37°C. Trophozoites in the log phase of growth were used in all experiments. For the glucose starvation assays, trophozoites in the exponential phase of growth were washed three times and transferred to Diamond's TYI-S-33 medium that has been prepared without glucose (glucose concentration 31 mg/l). Recovery from glucose starvation was done by direct addition of 1% glucose to the culture of starved parasites.

*Escherichia coli* strain BL21 (DE3): F^−^ ompT gal dcm lon hsdS_B_(r_B_^−^ m_B_^−^) λ(DE3 [lacI lacUV5-T7 gene 1 ind1 sam7 nin5])

*Saccharomyces cerevisiae* strain Y190: MATa, gal4 gal180 his3 trp1–901 ade2–101 ura3–52 leu2–3, −112 + ura3::GAL→lacZ, LYS2: GAL(UAS)→HIS3 cyh^r^

### DNA constructs used for:

#### Yeast two-hybrid screen

*An expression library of* random primed c-DNA from *E.histolytica* was prepared by Vertis Biotechnologie AG (Germany), and cloned in the pACT2 vector downstream to the GAL4 activation domain.

*Ehmeth* was amplified by PCR from *E. histolytica* genomic DNA using the primers Ehmeth Bam and Ehmeth3′ (Table 1), and then cloned in the pGEMT easy vector (Promega). The resultant vector was digested with BamHI and SalI, and the *Ehmeth* insert was then subcloned upstream to the GAL4 binding domain into the pAS1 plasmid that was previously linearized using BamHI and SalI (pGAL4-BD-Ehmeth).

**Table 1:** Primers used in this study.

Primer name	Sequence	Direction	Restriction site - underlined
Ehmeth kozak	GGATCCTAATACGACTCACTATAGGGAGCCACCATGGAACAGAAACAAGT	Sense	BamHI
Ehmeth 3′	TTATTCTTTTAAGTCATCGAATAAA	Antisense	
GST Enolase	GGCGGATCCATGTCAATTCAAAAGGTTC	Sense	BamH I
Enolase 3′	TATAGATCTTTAAGCAGTTGAATTTCTC	Antisense	Bgl II
Ehmeth(305)	TTAAATATTATAAATTTCTTTAAAAAC	Antisense	
Eno dro sma	ATCCCGGGAATGACCATCAAAGCGATCAAGG	Sense	Sma I
Eno dro	TTAAGCAGTTGAATTTCTCCAGTT	Antisense	
Ehmeth 265 kozak	GGATCCTAATACGACTCACTATAGGGAGCCACCATGTCTAAACATAAAGA	Sense	BamHI
Ehmeth 265 3′	ATAGATCTTATTGAATTATTATATGGTTGA	Antisense	Bgl II
Ehmeth 310 3′	TTAAATATTATAAATTTCTTTAAAAAC	Antisense	
DNMT2 Kozak	GGATCCTAATACGACTCACTATAGGGAGCCACCATGGTATTTCGGGTCTT	sense	BamHI
EhMRS2 5	GATTTTATTATATTTATTAATGTTTGA	sense	
EhMRS2 3	GATCCCATACAAAAATAATTACA	antisense	
Dnmt2 3′	ATAGCAAATATGTTGTATTTTGTTTTA	antisense	
Dnmt2 5′	ATGGTATTTCGGGTCTTAGAACTATT	sense	
Ehmeth Bam	TATGGATCCAACAGAAACAAGTAAATG	sense	BamH1
Enolase Bgl 3′	TATAGATCTTTAAGAGTTGAATTTCTC	antisense	Bgl II
Ehmeth start	ATGCAACAGAAACAAGTAAATGTTAT	sense	
Ehmeth kpn	TATGGTACCATGCAACAGAAACAAGTA	sense	KpnI
Ehmeth Bgl	TAGATCTCTTTTAAGTCATCGAATA	antisense	Bgl II
hDnmt2 Bam	GGCGGATCCATGGAGCCCCTGCGGGTGCT	Sense	
hDnmt2 3′	TTATTCATATAAGATTTTGATTAGT	Antisense	
hDnmt2 kozak	GGATCCTAATACGACTCACTATAGGGAGCCACCATGGAGCCACTGCGGGT	Sense	
dtRNA	TGGCGCCCAACGTGGGGCTC	Antisense	
T7	CGCGCGAAGCTTAATACGACTCACTATA	sense	
TNT Eno	GGATCCTAATACGACTCACTATAGGGAGCCACCATGTCAATTCAAAAGGT	sense	
Enolase kpn 5′	ATGGTACCATGTCAATTCAAAAGGTTC	sense	KpnI
Enolase NLS	GGATCCTTATCCAACCTTTCTTTTCTTTTTTGGTCCAGATCTAGCAGTTGAATTTCTCCAGTTCTTTCC	antisense	BamH1

#### *In vitro* translation

*Ehmeth* was amplified from *E.histolytica* genomic DNA by PCR using the primers Ehmeth start and Ehmeth3′ (Table 1), and then cloned in pGEM– T–easy vector (pGEMT-Ehmeth). In order to serve as DNA template in the in vitro translation assay (TNT), *Ehmeth* was amplified from pGEMT-Ehmeth by PCR using the primers Ehmeth Kozak and Ehmeth 3′ (Table 1).

Truncated Ehmeth 1–103 (from amino acid 1 to 103) was amplified from pGEMT-Ehmeth by PCR and the primers Ehmeth Kozak and Ehmeth 310 were used in order to serve as DNA template in the TNT system (Table 1). Truncated Ehmeth 88–322 (from amino acid 88 to 322) was amplified from pGEMT-Ehmeth by PCR using the primers Ehmeth 265 Kozak and Ehmeth 3′ (Table 1) in order to serve as DNA template in the TNT system.

The deletion of the 45 nucleotides that encoded Ehmeth amino acids 88–103 was done as follows: Ehmeth 1–88 was first amplified from pGEMT-Ehmeth by PCR using the primers Ehmeth start and Ehmeth 265 Bgl, and the PCR product was then cloned in pGEM–T–easy vector (pGEMT-Ehmeth1–88). Ehmeth 103–322 was amplified from pGEMT-Ehmeth by PCR using the primers Ehmeth310 Bgl and Ehmeth 3′, and the PCR product was cloned in pGEM– T–easy vector (pGEMT-Ehmeth103–322). The two plasmids, pGEMT-Ehmeth(88–322) and pGEMT-Ehmeth(1–88) were digested with Bgl II and EcoRI, and the Ehmeth DNA fragments were ligated using T4 DNA ligase (Biolabs). The product of the ligation was used as DNA template, and then amplified with the primers Ehmeth start and Ehmeth 3′. The resultant PCR product was cloned in a pGEM–T–easy vector (pGEMT-EhmethΔ(88–103)). EhmethΔ(88–103) was amplified by PCR using the primers Ehmeth Kozak and Ehmeth3′ in order to serve as DNA template for the TNT system.

The primers hDnmt2 5′ and hDnmt2 3′ were used for the amplification of hDnmt2 from a cDNA clone HGNC∶2977 (Open Biosystems) and then cloned in a pGEEM- T easy vector (pGEMT-hDnmt2) following for use as template in TNT system hDnmt2 was amplified from pGEMT-hDnmt2 by PCR with primers TNT- hDnmt2 and hDnmt2 3′.

#### Expression of recombinant proteins in *E.coli*

For the expression of the recombinant GST fusion proteins, Ehenolase was amplified from genomic DNA by PCR using the primers GST-Enolase and Enolase Bgl II 3′ (Table 1). The PCR product was then cloned in a pGEM-T easy vector (pGEM- Ehenolase), digested with BamHI and Not I, and then subcloned into the pGEX-4T1 vector (Amersham Pharmacia Biotech) that was previously linearized using BamHI and Not I. The preparation of Ehmeth-GST was done as previously described [12].

The primers hDnmt2-Bam and hDnmt2 3′ (Table 1) were used for the amplification of hDnmt2 from pGEM-hDnmt2. The PCR product was then cloned in a pGEM-T easy vector, digested with BamHI and Not I, and then subcloned into the pGEX-4T1 vector that was previously linerarized with BamH1 and Not I.

#### Expression of CHH tagged Ehmeth in *E.histolytica*

Ehmeth was amplified by PCR with the primers Ehmeth kpn and Ehmeth Bgl, and then cloned in the pJST4 expression vector (kindly provided by Prof. Lohia, Department of Biochemistry, Bose Institute, India) that was previously linearized with Kpn I and Bgl II. This vector allows the expression of a calmodulin binding domain, HA, His (CHH)-tagged protein in *E. histolytica* whose expression is driven by an actin promoter. The transfection of *E. histolytica* trophozoites was performed in the identical manner as previously described [52].

#### Expression of NLS enolase and NLS control in *E.histolytica*

Enolase was PCR amplified using primers Enolase kpn and Enolase NLS 3′ and cloned into the constitutive expression vector pEhNEO/CAT [53], which had been previously linearized by digestion with KpnI and BamHI. The pScramblePept3 plasmid that was previously used to express a scramble peptide fused to a NLS sequence in *E.histolytica* [30] was used as control. The transfection of *E. histolytica* trophozoites was performed as described in [52].

### Two hybrid analysis

*S. cerevesiae* Y190 was transformed with pGAL4-BD-Ehmeth (500 µg) using the LiAc transformation method [54].

The pGAL4-BD-Ehmeth strain was transformed with *E.histolytica* cDNA library (500 µg), and the transformants were then selected for their ability to grow on selective media that lacked leucine and tryptophan for four days at 30°C. After this first round of selection, the resistant clones were plated on a more selective media that lacked leucine, tryptophan, histidine, and adenine, and then grown for five days at 30°C. Fifteen resistant clones were then selected for further analysis. From these clones pACT2 vectors that contained cDNA inserts from *E.histolytica* library were isolated, and then transformed in the pGAL4-BD-Ehmeth strain. After the third round of selection, only two clones were able to grow on the selective media that lacked leucine, tryptophan, histidine, and adenine.

### *In vitro* transcription/translation

Coupled transcription and translation was carried out using a T7 TNT *in vitro* transcription/translation kit (Promega) in accordance with the manufacturer's instructions.

### Expression and purification of the recombinant proteins in *E. coli* BL21

For the expression of the different GST-recombinant proteins, *E. coli* BL-21 that were transfected with the corresponding vectors were grown overnight in Luria Broth (LB) medium that contained 100 µg/ml ampicillin. The pre-cultures were inoculated (1∶100) with 2xYT medium that was supplemented with 100 µg/ml ampicillin, and grown for about two hours at 37°C until the OD_600_ reached 0.8. Induction of the fusion protein was initiated by adding isopropyl-beta-D-thiogalactopyranoside (IPTG) at a final concentration of 0.5 mM to the growing culture. After a four-hour incubation at 30°C, the bacteria were harvested in lysis buffer (100 mM KCl, 1 mM DTT, 1 mM PMSF, 100 µg/ml Lysozyme and Leupeptine 100 µg/ml in PBS), and then sonicated for five minutes with 30 seconds of pulses with 30 seconds between each pulsation session. The lysis was completed by addition of BugBuster protein extraction reagent (1∶100) (Novagen). The recombinant GST-proteins were purified under native conditions on a gluthatione-agarose resin (Sigma). Aliquots of GST fusion proteins that were bound to the glutathione-agarose beads were conserved at −70°C for the pull-down assay. The remaining recombinant proteins were then eluted with glutathione elution buffer (Tris HCl 50 mM pH 8.0, glutathione (Sigma) 10 mM), and their concentration was measured by Bradford's method [55].

### GST pull-down assay

Gluthatione sepharose beads that were coated with GST- Enolase, or GST alone (20–50 µg) were incubated with in vitro translated [^35^S]-methionine-labeled proteins (15 µl of the TNT reaction) in a final volume of 500 µl pull-down buffer (20 mM Hepes pH 7.9, 100 mM NaCl, 1 mM DTT, 6 mM MgCl_2_, 20% glycerol, 1% Nonidet P40 and 0.5 mM EDTA) for one hour at room temperature. The beads were then centrifuged at 3000 rpm for five minutes, washed three times with the pull-down buffer, and then incubated at 100°C in presence of 25 µl Laemmli sample buffer for five minutes. Interacting proteins were resolved on 12% SDS-polyacrylamide gel electrophoresis or 15% SDS-polyacrylamide gel when TNT-Ehmeth (1–88) protein was used. The resultant bands were visualized after staining with Coomassie blue, drying and autography exposure.

### Trophozoites fractionation

*E.histolytica* trophozoites nuclear and cytoplasmatic fractions were prepared in the identical manner as previously described [24]. Proteins were resolved on 12% SDS-polyacrylamide gel electrophoresis, and then transferred to nitrocellulose membranes. Blots were then blocked (3% skim milk powder), and then reacted with either 1∶500 enolase antibody (Santa Cruz Biotechnology) or with 1∶500 HA antibody (Santa Cruz Biotechnology). After incubation with the first antibody, the blots were incubated with 1∶5000 corresponding second antibody (Jackson ImmunoResearch), and then developed by enhanced chemoluminescence.

### Determination of pyruvic acid in the lysate of control and glucose-starved trophozoites

Trophozoites (10^6^) that were grown in regular or glucose-deficient media were washed three times with PBS, and then resuspended in 2 ml of PBS. The trophozoites were lysed by freezing and then thawing to produce a total protein lysate. The pyruvic acid level in trophozoite lysates was determined according to a previously described method [56]. Briefly, 1 ml of 2,4- dinitrophenylhydrazine (DNPH) (0.0125% in 2 N HCL) was added to 1 ml of trophozoite lysate. After 15 minutes of incubation at 37°C in a water bath, the sample was removed from the water bath, and 5 ml 0.6 N NaOH was added. The absorbance of the sample was then measured in a spectrophotometer at 420 nm. A standard curve was generated using sodium pyruvate [56].

### Production of anti-enolase antibody

Male BALB/c mice were injected intraperitoneally with 100 mg of GST-Enolase recombinant protein that was emulsified in complete Freund's adjuvant. Two and four weeks later, the mice were injected with 100 mg of the recombinant protein in incomplete Freund's adjuvant. One week after the 4-week injection, about 0.8 ml of sera was obtained by retro-orbital puncture. Serum that was obtained from mice that were not injected with recombinant protein was used as the control.

### Microscopic localization of enolase in trophozoites

Trophozoites in a logarithmic growth phase were harvested, transferred to 8 mm round wells on glass slides, and then incubated for 30 min at 37°C in order to allow them attach to the glass surface. An indirect immunofluorescence assay was performed. For this purpose, the amebae were fixed with cold methanol for 20 min at −20 C, and then incubated with 1∶400 enolase antibody for one hour at room temperature. After washing, the samples were then incubated with goat Cy3-conjugated anti-mouse (Jackson ImmunoResearch) 1∶1000 for one hour. Samples were then stained with 4,6-diamidino-2-phenylindole dihydrochloride (DAPI,Sigma) in order to visualize the nuclei. Fluorescent images were captured by a CCD camera attached to an Axioscop2 (Zeiss) epifluorescence microscope with a 100/1.30 Plan Neofluar oil immersion objective and a differential interference contrast filter. The images were analyzed with ImagePro@Plus software (Media Cyberneticx, USA).

### Immunoprecipitation assays

Aliquots of nuclear protein fraction (50 µg) were diluted in 20 mM Hepes pH 7.5, 150 mM NaCl, 0.1% Triton, 10% glycerol (HNTG buffer) (300 µl), and then incubated with protein G beads (Sigma) (10 µl) for 30 minutes at 4°C. Non-specific interacting proteins were excluded by centrifugation (3000 rpm at 4°C for 5 minutes). The supernatant was incubated with either 1∶200-HA antibody or enolase antibody) for two hours at 4°C. Following incubation protein G-Sepharose beads (20 µl) were added to the samples which were then incubated for 16 hours at 4°C. Immunoprecipitated proteins were collected by centrifugation, washed three times with HNTG buffer, and then resolved by 12% SDS-polyacrylamide gel electrophoresis. The proteins were then transferred to nitrocellulose membranes by western blot analysis, and detected with the relevant antibody, mouse anti-enolase or rabbit anti-HA.

### Preparation of the EhMRS probe

EhMRS2 was amplified from *E.histolytica* genomic DNA by PCR and the primers EhMRS2 5 and EhMRS2 3. EhMRS2 DNA (10 pmol) was end-labeled with T4-polynucleotide kinase (New England Biolabs) and γ-ATP in accordance with the manufacturer's recommendations. Unincorporated γ-ATP was removed with the ProbeQuant kit (Amersham).

### Examination of the effect of enolase on the binding of Ehmeth and hDnmt2 to EhMRS2 DNA

Gluthatione sepharose beads that were coated with GST-Ehmeth, GST-hDnmt2, or GST alone (35 µg) were incubated in 100 µl blocking buffer (3% BSA and salmon sperm DNA 1 µg/ml in standard binding buffer (20 mM Tris-HCl pH 8, 50 mM NaCl, 1 mM EDTA in double distilled water) for 30 minutes at room temperature. Following blocking the beads were washed three times with standard binding buffer, and incubated with either 40 µg or 60 µg of GST- Enolase for one hour at room temperature (100 µl final reaction volume). The probe (0.3 µg) was then added, and binding was carried out at 4°C overnight. Subsequently, the beads were washed three times in standard binding buffer, boiled with 25 µl Laemmli sample buffer for 5 minutes; proteins were separated on 10% SDS-polyacrylamide gel electrophoresis. The signal of the proteins that were bound to the labeled DNA probe was detected directly from the polyacrylamide gel on X-ray film (Fuji).

### tRNA preparation

The methylation assay of tRNA^Asp^ with the DNMT2 variants was performed using a previously described method [6]. Briefly, the DNA template that encoded Drosophila tRNA^Asp^ was amplified by PCR and the T7 primer and tRNA^Asp^ primer. For *in vitro* transcription, 100 µl of the PCR reaction were incubated with 200 µl 2× transcription buffer (80 mM Tris-HCl at pH 8.1, 2 mM Spermidine, 10 mM DTT, 0.02% Triton-X-100, 60 mM MgCl2, 4 µg/ml BSA), 5 mM of each NTP (final concentration), and 10 µl of T7-Polymerase (200 units/µl; Fermentas) in a final volume of 400 µl for three hours at 37°C. Transcripts were purified over 12% denaturing PAGE, and bands of correct size were excised, eluted in 0.5 M ammonium acetate, and precipitated with two volumes of 100% ethanol. After centrifugation, RNA pellets were washed once with 80% ethanol, and then dissolved in double distilled water. The concentration of tRNA was measured with a nanodrop spectrophotometer.

### Matrix-assisted laser-desorption/ionization – time of flight (MALDI-TOF) mass spectrometry analysis

Protein bands of interest were excised from the SDS-polyacrylamide gel and digested with trypsin using a previously published protocol [57], and then analyzed by MALDI-TOF mass spectrometry analysis that was done at the Institute of Biology, Technion, Israel. The peptide mass profiles that were produced by MALDI-TOF mass spectrometry were processed using PepMiner (this software is described at http://www.haifa.il.ibm.com/projects/verification/bioinformatics/). Peptides masses were compared with the theoretical masses that were derived from the sequences that were in the SWISS-PROT/TrEMBL (http://www.expasy.ch/sprot/), the NCBI (http://www.ncbi.nlm.nih.gov/), and the *E. histolytica* genome project databases (http://pathema.jcvi.org/cgi-bin/Entamoeba/GenomePage.cgi?org=eha2).

### *In vitro* tRNA methylation assay

Aliquots (40 pmol) of Drosophila tRNA^Asp^ were incubated with 0.4 nmol Ehmeth or 0.04 nmol GST-hDnmt2 for three hours at 37°C in 40 µl of methylation buffer (100 mM Tris/HCl at pH 7.5, 5% glycerol, 5 mM MgCl2, 1 mM DTT, and 100 mM NaCl) that contained 4.2 µM labeled [methyl-3H] AdoMet (NEN). When we examined the effect of enolase on Ehmeth activity, GST-Enolase (2 nmol) or GST as negative control (2 nmol) were incubated with Ehmeth for one hour at 37°C. When we examined the effect of enolase on hDnmt2 activity, GST-Enolase (0.4 nmol) or GST (0.4 nmol) were incubated respectively with hDnmt2 for one hour at 37°C. Samples (8 µl) were taken from reaction mix (40 µl) at different times, and loaded on Whatman filters. The filters were then washed with 10% Trichloroacetic Acid Solution (TCA) three times and finally with 100% ethanol. After washing the filters were air-dried and transferred into tubes following addition of 3 ml scintillation liquid (CytoScint). The incorporated radioactivity was measured in a scintillation counter (Counter Beta Tri-Carb 2100TR). tRNA methyltransferase activity (one unit (U)) was expressed as the incorporation of 1 pmol AdoMet per hour per nmol of protein. *In vitro* tRNA methylation assay in the presence of 2 phosphoglycerate (2-PG) (Fluka) was done in the identical manner with minor modifications. Increasing 2-PG concentrations (1–7 mM) were incubated with GST-Enolase (0.4 nmol) and with hDnmt2 (0.04 nmol). The activity of hDnmt2 in the presence of 7 mM 2-PG was used as control.

### *In vitro* methylation assay of total RNA

Total RNA was prepared with the TRI-Reagent kit (Sigma) from control or glucose-starved trophozoites and treated with DNase I to remove any contamination of DNA. Aliquots from the treated RNA (20 µg), were used as substrates for hDnmt2 in vitro tRNA methylation assay (see above protocol). The amount of methyl groups that was incorporated by hDnmt2 into the tRNA of each sample is proportional to the amount of unmethylated tRNA in the control sample.

### Accession numbers of genes and proteins mentioned in the text

*E.histolytica* enolase: XP_649161.1, Ehmlbp: XP_649236, Ehmeth: XP_655267.2, Myosin II: XM_651936.1, hDNMT2: NP_004403.1

## Supporting Information

Figure S1 Mass spectrometry analysis of the retarded band observed following incubation of Ehmeth with EhMRS2 DNA.
(0.49 MB TIF)
