# Supplementary figures and images for: A New Nuclear Function of the Entamoeba histolytica Glycolytic Enzyme Enolase: The Metabolic Regulation of Cytosine-5 Methyltransferase 2 (Dnmt2) Activity
Source: PLoS Pathog. 2010 Feb 19;6(2):e1000775. doi: 10.1371/journal.ppat.1000775 (PMC2824750; doi:10.1371/journal.ppat.1000775)

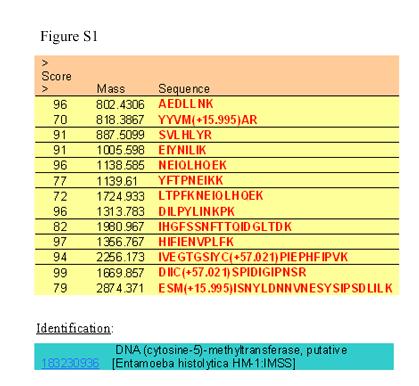

Supplement: Figure S1 — Mass spectrometry analysis of the retarded band observed following incubation of Ehmeth with EhMRS2 DNA. (0.49 MB TIF) [file ppat.1000775.s001.tif]
